# Experimental demonstration of topological effects in bianisotropic metamaterials

**DOI:** 10.1038/srep22270

**Published:** 2016-03-03

**Authors:** Alexey P. Slobozhanyuk, Alexander B. Khanikaev, Dmitry S. Filonov, Daria A. Smirnova, Andrey E. Miroshnichenko, Yuri S. Kivshar

**Affiliations:** 1 Nonlinear Physics Center, Australian National University, Canberra ACT 2601, Australia; 2 Departament of Nanophotonics and Metamaterials, ITMO University, St. Petersburg 197101, Russia; 3 Department of Physics, Queens College of City University of New York, NY 11367, USA; 4 Department of Physics, Graduate Center of City University of New York, NY 10016, USA

## Abstract

Existence of robust edge states at interfaces of topologically dissimilar systems is one of the most fascinating manifestations of a novel nontrivial state of matter, a *topological insulator*. Such nontrivial states were originally predicted and discovered in condensed matter physics, but they find their counterparts in other fields of physics, including the physics of classical waves and electromagnetism. Here, we present *the first experimental realization* of a topological insulator for electromagnetic waves based on engineered *bianisotropic metamaterials*. By employing the near-field scanning technique, we demonstrate experimentally the topologically robust propagation of electromagnetic waves around sharp corners without backscattering effects.

Topologically nontrivial states of light^1^ represent a “Holy Grail” for optical applications usually suffering from undesirable backscattering and interference effects, thus dramatically limiting the bandwidth and performance of many photonic devices. Recently there were numerous *theoretical predictions* of topologically nontrivial states in different photonic systems ranging from microwave to optical frequencies^2^,^3^,^4^,^5^,^6^,^7^,^8^,^9^,^10^, but experimental demonstrations are very limited. In particular, the topological protection was shown experimentally for gyromagnetic photonic crystals^11^,^12^, arrays of coupled ring resonators^13^,^14^, chiral optical waveguides^15^, and microwave waveguides^16^. In all such systems, different approaches were suggested to emulate an effective magnetic field, in a direct analogy with the familiar Quantum Hall Effect (QHE). However, there exists another class of electromagnetic systems, for which topological nontrivial states emulating Quantum Spin Hall Effect (QSHE)^17^,^18^,^19^,^20^,^21^,^22^ and robust helical edge states were predicted being based on bianisotropic metamaterials^9^,^10^ - a special class of synthetic periodic optical media with engineered magnetoelectric response^23^,^24^,^25^,^26^. Here we suggest and demonstrate the first experimental realization of a topologically nontrivial metamaterial which emulates directly *spin-orbit coupling in solids*, and also exhibits a desirable property of topologically robust edge transport enabling guiding waves through sharp corners. We emphasize here that our design represents *a true open metacrystal* which, in a sharp contrast to the waveguide geometries employed earlier^10^,^16^, enables a direct mapping of the near-field of the topological edge states.

Topological electromagnetic states realized in our system are based on two key ingredients: (i) “spin-degeneracy” enabling emulation of the electron spin, and (ii) bianisotropic gauge fields mimicking the spin-orbital coupling for electrons in solids. We consider a metacrystal with a square lattice of “metamolecules” with the shape of a ring and two symmetric slits and a wire placed in the middle. A schematic of the single metamolecule and the periodic arrangement of the metacrystal are shown in Fig. 1. The metamolecule of this geometry possesses two low-frequency magnetic and electric dipolar resonances with their field profiles shown in Fig. 1b. The magnetic resonance is formed by the antisymmetric charge distribution in the wires of the split ring and with the inactive central wire (an upper panel in Fig. 1b). The magnetic dipole moment of this mode is oriented in the direction orthogonal to the plane of the metamolecule, and it originates from the counter-propagating current in the wires. It is also easy to see that the net electric dipole moment of this mode vanishes due to cancellation of the dipole moments of the individual antennas of the split ring. In contrast, the electric dipolar resonance originates from the dipole moment of the central wire modified by the interaction with the wires of the split ring. This resonance appears to be shifted significantly towards the subwavelength range due to the hybridization with the split-ring by inducing symmetric-current distribution in the wires. The latter feature allows us to satisfy the spin-degeneracy condition as the geometrical parameters of the metamolecule can be tuned to ensure that the electric and magnetic dipolar resonances occur at the same frequency.

As the next step, we create a metacrystal by arranging the metamolecules into a square two-dimensional array with only one layer in vertical direction (see Fig. 1c). The open geometry considered here represents a special interest due to possible leakage of topological modes caused by the out-of-plane scattering into the radiative continuum. The square lattice of the metacrystal with 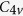 in-plane and z-inversion symmetries possesses quadratic degeneracies at high symmetry Γ and M points in the Brillouin zone^4^,^12^,^27^, which is confirmed for both electric and magnetic dipolar modes of the metacrystal by first principle finite element method (FEM) simulations (see Methods for details), and are shown in Fig. 1d (green dotted line). Here we are interested in guided waves exponentially confined to the structure in vertical (z-) direction and whose bands are located below the light cone (dashed straight lines in Fig. 1d), and, therefore, in what follows we focus on the quadratic degeneracies taking place at M points. Note, that to ensure the condition of spin-degeneracy at M-point, e.g. the two doubly-degenerate quadratic bands stemming from magnetic and electric resonances appear at the same frequency, we fine-tuned the geometry of the metamolecules to compensate for the effects of slightly different interaction between magnetic and electric dipoles in the array. The band structure (see Fig. 1d) confirms the presence of overlaid electric-like and magnetic-like collective modes with quadratic degeneracy at the M point.

The presence of the quadratic degeneracies is crucial for topological protection in the metamaterial under study; to illustrate this, here we first apply the analytic effective Hamiltonian description^3^,^9^,^27^. The band structure of the metacrystal in the proximity of the M-points can be described by the effective quadratic Hamiltonian 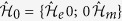, acting on a four component wavefunction 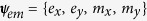, with its components being in-plane electric and magnetic dipole moments, and subscripts *e* and *m* indicating the electric and magnetic blocks, respectively, which are described by the expression^27^:


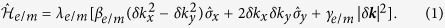


Here 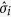 are the Pauli matrices, 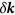 is the deviation of the Bloch wavevector from the M-point, and 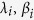, and 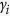 are the effective parameters of the model, which in general differ for electric and magnetic modes. However, in our design the dispersion of the electric and magnetic modes has been tuned to match near the M-point. This degeneracy is crucially important as it allows choosing a new set eigenmodes as any linear combination of electric and magnetic components. One of the key ingredients for engineering the topological state in the metacrystal represents the ability to emulate the spin degree of freedom of electrons in QSHE, which must be odd under time reversal (TR) operation. By noticing that electric and magnetic moments transform differently under TR 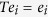 and 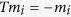, it was shown that an appropriate choice of basis is given by the wavefunction 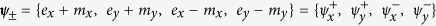, where 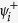 and 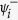 are the pseudo-spin components which are time-reversal partners 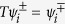 of each other^9^.

Next, to endow the metacrystal with topological properties, we introduce an effective gauge field emulating the spin-orbital coupling, which is achieved by introducing bianisotropy mixing magnetic and electric degrees of freedom of the system^9^. The desirable form of bianisotropy should couple x-(y-) magnetic dipole moment with y-(x-) electric dipole moment of the metamolecule, and is realized by breaking its z-inversion symmetry^23^. Figure 1d (red dotted line) shows the band structure calculated by the FEM for the case of distorted metamolecules, where the central wire was displaced vertically between the split ring wires (so that gaps 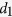 and 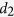 are no longer equal), and reveals a gap that is complete below the light cone. It is important to note, that the four-fold degeneracy around the M-point does exist in ideal square lattice of resonant elements, as dictated by the lattice symmetry. The small opening of the gap within electric-like and magnetic-like modes is, in fact, induced by the presence of the dielectric substrate, which induces coupling between these modes, however, it appears to be very narrow in comparison with the topological bang gap open by the bianisotropy and, according to our simulations and experimental results, has no effect on the edge states.

The presence of a non-zero bianisotropy, playing the role of spin-orbit coupling, which in the basis of electric and magnetic dipolar modes can be described by the effective potential 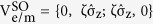 with the real parameter 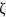 characterizing the strength of the bianisotropy, leads to the removal of this degeneracy^9^. The four bands near the point of quadratic degeneracy thus are described by the 4 × 4 eigenvalue problem


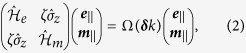


where 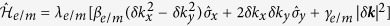, 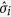 are the Pauli matrices, 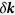 is the deviation of the Bloch wavevector from the M-point, and 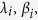 and 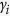 are the effective parameters of the model^27^.

When the condition of spin-degeneracy is satisfied (e.g. 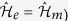, and the degenerate quadratic bands for electric and magnetic modes appear at the same frequency, the equation (2) can be block diagonalized by the linear transformation 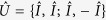 to the spin-up/spin-down basis 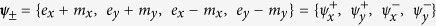 9:


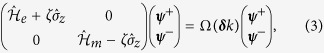


where 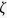 is now clearly playing the role of the mass term which has an opposite sign for the spin-up and spin-down states^9^,^27^.

By solving the eigenvalue problem (3), we find the bulk band structure of the metacrystal, which appears to be doubly degenerate with respect to the spin:


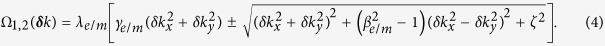


As can be seen from Eq. (4), for 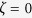 the two quadratic bands meet at 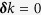, for both spin-up 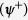 and spin-down 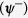 states, while at 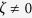 the degeneracy is lifted and a bulk bandgap opens leading to an “insulating” state, which is illustrated in Fig. 2(a,b) by black solid lines.

As the next step, we focus on the edge states at a domain wall located at 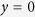 between two locally homogeneous regions, where the sign of the bianisotropy parameter 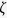 (and the mass term) reverses. Edge-state solutions propagating along the domain wall but decaying in the direction perpendicular to the domain wall will lie in the bulk gap of the two domains^28^. Since the Hamiltonian is of the second order in the momenta, the dispersion relation for edge states are found by imposing the boundary conditions to the wave functions which require the continuity of the wave function and the absence of the current normal to the edge. The resultant dispersion of the edge modes found both for spin-up and spin-down states is shown in Fig. 2 by red and blue solid lines, respectively.

The sign of the mass parameter 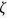 in the effective Hamiltonian is clearly related to the topological invariants associated with the non-trivial topological character of the photonic states in the proposed metamaterial design. Particularly, calculating the Chern numbers for the upper and lower bands 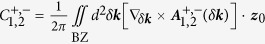 by integrating the Berry connection 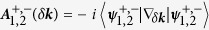 expressed through the eigenvectors 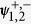 of the problem (3) gives 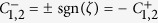
27. Thus, although the net Chern number for a pair of frequency-degenerate Kramers partners vanishes because of the time-reversal symmetry, the metacrystal exhibits a topologically non-trivial phase characterized by the spin Chern number 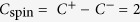 and an invariant 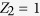
18, which is analogous to the case of electronic quantum spin Hall system.

Appearance of topologically robust helical edge modes insensitive to local structural imperfections and avoiding backscattering represents a hallmark of topological photonic order emulating QSHE^8^,^9^,^29^. In the case of bianisotropic metacrystals^9^, the edge states have been predicted to occur at the domain walls representing boundaries between regions of the metacrystal with the reversed bianisotropy. The presence of such modes was confirmed by calculating the photonic band structure in the effective Hamiltonian model. From the bulk-boundary correspondence, for such a domain wall, one expects two edge modes for every spin, in accordance with the change in the spin-Chern number between the domains^9^. It is worth mentioning that one could also expect to observe the edge modes on the external boundary of the metacrystal with the air, however, in contrast to electronic systems, the external domain does not possesses a bandgap in the spectrum and is filled with the electromagnetic continuum. This makes such modes either leaky or unstable to any perturbation which couples them to the radiative continuum of the free space, thus breaking the topological protection.

To verify these theoretical predictions and confirm topological robustness of the helical edge states, a metacrystal was fabricated by printing an array of metamolecules on the 1 mm-thick dielectric board (Arlon 25N) with the dielectric permittivity 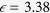. The board was cut into linear segments which were stacked to form a square lattice, as shown in Fig. 3a. To test the topological protection, which leads to the ability of the edge modes to propagate around sharp bends without back scattering^9^, the zigzag-shaped domain wall was created by deliberate distribution of metamolecules with the central wires shifted vertically up and down across the crystal, as indicated by the dashed white line in Fig. 3a.

First, the presence of the topological band gap was verified by exciting bulk modes with the dipole source placed away from the domain wall. Figure 3b clearly reveals the gap spanning frequency range from 8.6 GHz to 9.3 GHz. Next, the presence of the topological edge mode was tested by placing the dipole at the domain wall and the transmission spectrum was measured. As expected, the enhanced transmission within the band gap region occurred due to the excitation of the edge mode, as shown in Fig. 3b. Note that the transmission was especially high near the gap center and gradually dropped towards the edges of the bands.

To confirm that the transmission indeed occurs due to the edge mode localized to the domain wall, the near-field map of magnetic field intensity was measured across the entire sample with the use of the probe (magnetic loop antenna) mounted on the two-dimensional near-field scanning stage. The map shown in Fig. 3c clearly shows that the mode excited at the frequency of 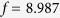 GHz is indeed guided by the domain wall. In agreement with the theoretical predictions, the mode appears to be strongly localized to the domain wall. In addition, quite surprisingly we have not noticed any apparent effects of leakage neither to the modes above the light cone nor to the radiative continuum. Moreover, we observed that regardless of the open character of the system, the edge modes do not scatter to these modes even when they encounter sharp bends of the domain wall, and the wave flawlessly transfers between orthogonal segments of the wall. The mode is not radiating to the free space at the sharp corner due to its strong localization to the domain wall and the spin locking, as well as the profile mismatch with the modes above the light cone. Indeed, the mode simply has higher quality factor and the energy tends to stay in this mode rather than being transferred into the short-living leaky state near the gamma point. To ensure the robustness across the entire frequency range of the topological bandgap, the same measurements have been conducted for multiple frequencies. As expected for the frequencies close to the spectral edges of the bulk modes, the edge states become poorly localized and are hardly distinguishable from the bulk modes. However, as can be seen from Fig. 3d, the edge states with frequencies sufficiently apart from the bulk spectrum remains well localized and exhibits similar robustness against sharp bends of the domain wall at all frequencies. It is also important to mention that the drop in the transmission observed in Fig. 3b away from the center of the topological band gap lacks back-reflection along the domain wall (observed in Fig. 3d), which indicates that the low transmission near the edges is associated with the insertion loss. This can be explained as the consequence of increasingly poor overlap of the field produced by the dipole antenna with the field profile of the edge mode which spread over the bulk as we approach the band edges. Also note that in Fig. 3d some variations in the field intensities occur due to the local interference effects, which, however, do not lead to backscattering, but rather assist total transmission through the zigzag, as is evidenced by the rebound of the intensity away from the 90°-bends of the wall. It should be mentioned that the field profile in Fig. 3d indicates that there is slow decay of the field with the rate of about 12% per one passage through the structure. Nonetheless, this level of loss in our system is clearly marginal and does not affect the topological nature of the edge states. Finally, it’s important to mention that according to our measurements the finite lateral size of the sample did not play any detrimental role and no any noticeable backscattering of the edge modes occurred at the metacrystal boundaries, where the edge modes were efficiently radiated into the propagating continuum of the free space.

Topological robustness of electromagnetic helical edge-states in an open metamaterial system demonstrated here for the first time can be of significant interest both from fundamental and applied perspectives. First, it confirms the possibility to emulate exotic quantum states of solids in open metamaterial structures thus allowing direct near-field mapping of the local amplitudes and phases of topological waves. This enables the study of their topological characteristic and peculiarities in the real coordinate space as well as in the reciprocal space by performing Furrier transformation of the measured field maps. Second, the possibility to guide electromagnetic energy around arbitrarily shaped pathways avoiding undesirable back-reflection from sharp bends and without leakage into the radiative continuum brings the versatility of control of the electromagnetic energy flows in engineered photonic systems to an unprecedented level. It is only a matter of time when the full potential of topological photonic states will be exploited to study novel fundamental electromagnetic phenomena and in applied systems and devices with their physics and functionality solely relaying on the presence of the photonic topological order.

## Methods

### Numerical tools

**Figure 1:**
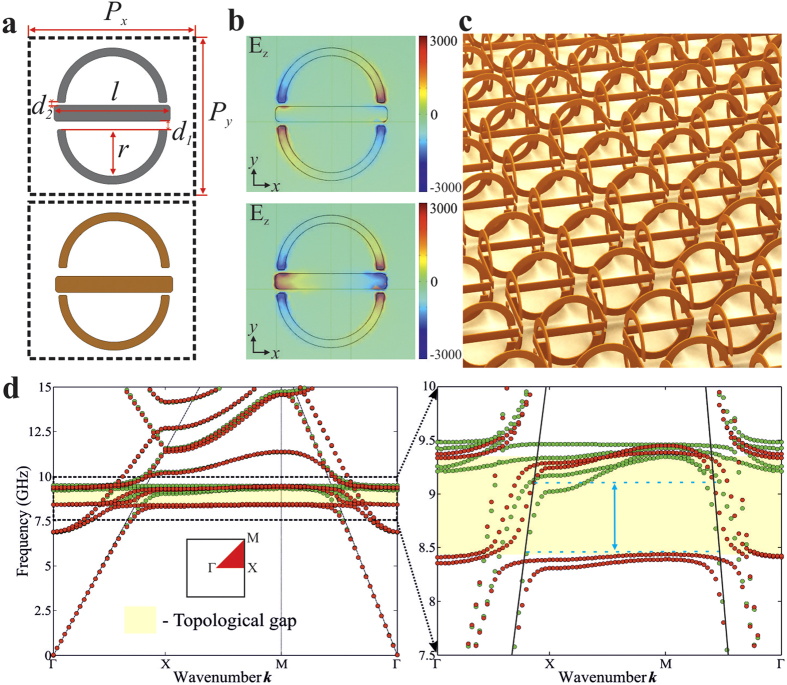
Structure and eigenmodes of the bianisotropic metamolecules and a metacrystal. (**a**) Geometry of the metamolecules with bianisotropic response controllable by the asymmetry of 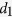 and 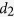 gaps. The radius (*r*), length of the wire (*l*), and periods (*P*_*x*_, *P*_*y*_) are equal to 2.85 mm, 0.5 mm, 7.5 mm, 13 mm and 12 mm respectively. Metal width for the central wire and split ring wires is 1 mm and 0.5 mm, respectively. (**b**) Dipolar magnetic (top subplot) and dipolar electric (bottom panel) eigenmodes of the symmetric metamolecule 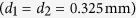. (**c**) Perspective view of the two-dimensional metacrystal formed by periodic stacking of the metamolecules. (**d**) Photonic band structure of the metacrystal with 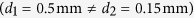 and without 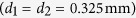 bianisotropy shown by red and green markers, respectively. An inset shows the first Brillouin zone and the high symmetry points. The right sub-image shows an enlarged region near the topological band gap. The yellow shaded area illustrates the spectral width of the gap and two blue lines indicate the position of the complete band gap region. The substrate with the permittivity 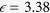 is taken into account. The position of the light line 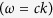 is marked by thin black dashed (on the left subplot) and solid (on the right subplot) lines.

All numerical results for photonic band structures are obtained by performing finite-element-method calculations in COMSOL Multiphysics (Radio Frequency module). We assume perfectly conducting elements (split ring and wire) on the dielectric substrate. The periodic boundary conditions are imposed in the *x* and *y* directions to form an infinite square lattice (shown in Fig. 1c). The perfect matched layers are added to the domain in order to prevent back reflections from *z* direction. The mesh is optimized in order to reach the convergence.

### Experimental approach

**Figure 3:**
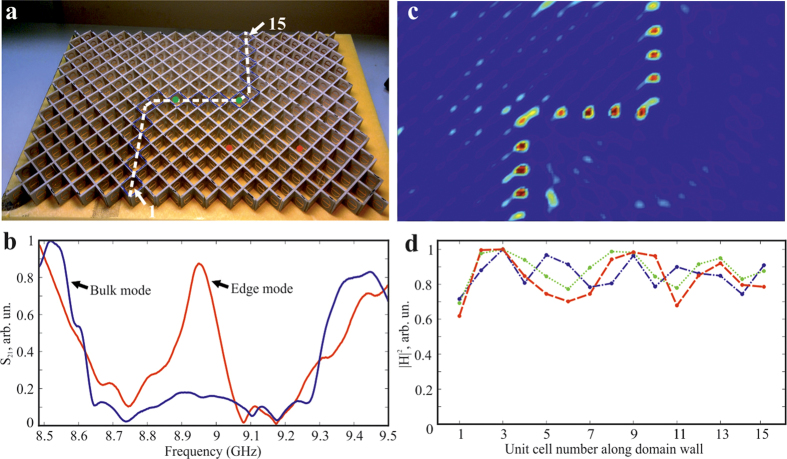
Experimental system and the observation of topological edge modes. (**a**) Photograph of the fabricated metacrystal with the location of the double-bend domain wall indicated by the white dashed line. (**b**) Transmission spectra of the metacrystal away from the domain wall (blue line - the dipoles locations are indicated by red circles on the panel (**a**) and along the domain wall (red line- the dipoles locations are indicated by green circles on the panel (**a**). (**c**) Two-dimensional map of the magnetic field intensity indicating reflection-less propagation along the domain wall. (**d**) Normalized magnetic field intensity along the domain wall measured for different frequencies (8.9 GHz - blue line; 8.95 GHz - green line; 8.987 GHz - red line) within the topological band gap.

The metamolecules are fabricated on the dielectric board (Arlon 25N) using chemical etching technique. Subsequently, the board is cut into linear segments which are staked to form a square lattice. Special mask made from styrofoam material with the dielectric permittivity of 1 is used to control the periodicity of the segments separation. All the measurements are performed in the anechoic chamber. We utilize subwavelength dipole as a source, which is connected to the transmitting port of a vector network analyser (Agilent E8362C). For measurement of the transmission spectra shown in Fig. 3b, a similar dipole antenna is used as a receiver. To perform the near field measurements we use an automatic mechanical near-field scanning setup and a magnetic field probe connected to the receiving port of the analyser (Fig. 3c). The probe is oriented normally with respect to the interface of the structure. The near field is scanned at the 1 mm distance from the back interface of the metacrystal to avoid a direct contact between the probe and the sample.

## Additional Information

**How to cite this article**: Slobozhanyuk, A. P. *et al*. Experimental demonstration of topological effects in bianisotropic metamaterials. *Sci. Rep*. **6**, 22270; doi: 10.1038/srep22270 (2016).

## Figures and Tables

**Figure 2:**
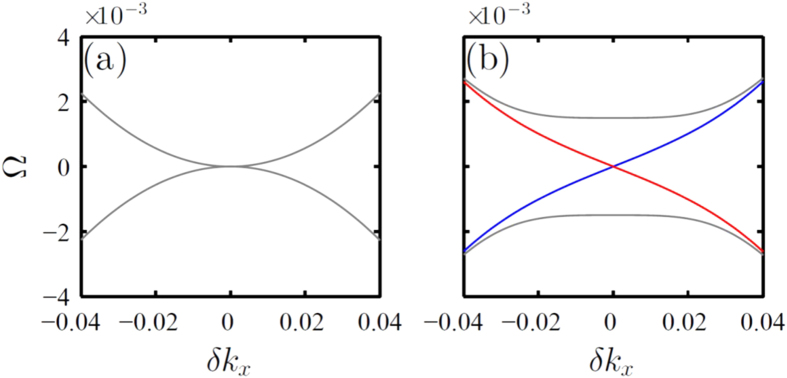
Bulk and edge modes of the metacrystal with a square lattice as described by the effective Hamiltonian. (**a**) Gapless bulk spectrum calculated for 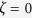 (total four bands are present, and spin-up and spin-down modes are doubly degenerate for all values of 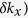. (**b**) Opening of the doubly-degenerate topological gap (black lines) and emergence of the spin up (red line) and spin down (blue line) edge states. Parameters used are 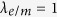, 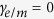, 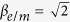, and 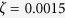.
